# Parental smoking and risk of hypospadias: An updated meta-analysis of observational studies

**DOI:** 10.3389/fped.2023.1003037

**Published:** 2023-02-23

**Authors:** Zi-Han Ye, Hong-Song Chen, Zhi-Cheng Zhang, Xiao Wang, Xing Liu, Guang-Hui Wei

**Affiliations:** ^1^ Department of Urology, Children’s Hospital of Chongqing Medical University, Chongqing, China; ^2^ Ministry of Education Key Laboratory of Child Development and Disorders, National Clinical Research Center for Child Health and Disorders, China International Science and Technology Cooperation Base of Child Development and Critical Disorders, Chongqing, China; ^3^ Chongqing Key Laboratory of Children Urogenital Development and Tissue Engineering, Chongqing, China

**Keywords:** hypospadias, maternal active smoking, maternal passive smoking, paternal smoking, and meta-analysis

## Abstract

**Background:**

Inconsistent relationships have been shown between cigarette smoking and hypospadias in offspring. The purpose of this study was to summarize epidemiological evidence to evaluate the relationship between parental smoking and the risk of hypospadias.

**Methods:**

Up until October 2022, PubMed, EMBASE, Web of Science, and the Cochrane Library were systematically searched for qualified research. The summary RRs and 95% CIs were calculated using either a fixed-effects or a random-effects model. There were subgroup analyses undertaken to identify potential sources of heterogeneity.

**Results:**

44 studies with 16,637,830 participants were included in our meta-analysis. Overall, maternal active smoking [risk ratio (RR) = 0.94; 95% confidence interval (CI): 0.90–0.99; *P* < 0.01] was significantly associated with the risk of hypospadias. And neither paternal smoking (RR = 1.00; 95% CI: 0.86–1.15) nor maternal passive smoking (RR = 0.91; 95% CI: 0.60–1.23) was associated with the risk of hypospadias.

**Conclusion:**

Our study discovered an association between maternal active smoking and a decreased risk of hypospadias, which may be due to the effect of smoking on androgen. However, as numerous studies have proved that cigarette smoking during pregnancy increases the risk of overall birth abnormalities in offspring, quitting cigarettes before pregnancy positively influences the health of offspring and should be advocated worldwide.

**Systematic review registration:**

[www.crd.york.ac.uk/prospero], identifier [CRD42022319378].

## Introduction

Hypospadias is a male urogenital congenital defect characterized by a disturbed urethral fusion between gestational weeks 8 and 14, which locates the urethral meatus ventrally from the penile glans to the scrotum or perineum (1). It occurs in roughly 0.5 percent of live newborns (2). However, the risk factors and the etiology of hypospadias remain controversial.

Extensive clinical and laboratory studies on the biological mechanisms by which tobacco smoke affects fetal development have revealed that many of the 7,000 compounds can cross the placental barrier and have adverse effects on the developing fetus (3–5). Moreover, smoking during pregnancy seems to be associated with various congenital abnormalities, such as gastrointestinal abnormalities, eye defects, digit anomalies, oral clefts, limb abnormalities, musculoskeletal deformities, heart defects, and cryptorchidism (6). Even though smoking during pregnancy increases the risk of most congenital defects, many pregnant women continue to smoke (7).

A previous meta-analysis of 15 studies on this topic revealed that pregnant women who smoke have a decreased risk of hypospadias (6), while a recent meta-analysis of 12 studies indicates that pregnant women who smoke have an increased risk of hypospadias (8). However, the number of studies included in these two meta-analyses is quite limited. Moreover, despite the fact that paternal smoking and maternal passive smoking are more common, these two meta-analyses did not conduct a meta-analysis of the association between maternal passive smoking or paternal smoking and the risk of hypospadias. Numerous studies evaluating the risk of hypospadias associated with paternal active smoking and maternal passive smoking produced equivocal findings.

Notably, a number of studies, including a large number of cohort studies with adequate sample sizes, have been published, which will help provide an adequate number of studies to assess the risk of hypospadias with greater confidence and to investigate plausible causes for heterogeneity. Prior meta-analyses did not consider the duration of smoking exposure. Early in pregnancy, the important phase of fetal urethral development occurs. Examining the relationship between parental smoking in early pregnancy and hypospadias is crucial, as this may help explain causation.

Therefore, with newly accumulating evidence, we aimed to conduct a meta-analysis with two aims: (i) to determine the risk of hypospadias associated with parental smoking, including maternal active smoking, paternal active smoking, and maternal passive smoking; and (ii) to identify potential moderators of heterogeneity using subgroup, including subgroups based on smoking exposure time, and other potential factors.

## Material and methods

### Search strategy

This research followed PRISMA standards and was pre-registered with PROSPERO: CRD42022319378, outlining our investigation's objectives and procedures.

### Information sources

This meta-analysis included articles published before October 23, 2022, about maternal or paternal smoking and the risk of hypospadias. Using the Boolean approach, relevant papers were retrieved from PubMed, EMBASE, Web of Science, and the Cochrane Library. The following search terms were utilized in the systemic search: (smoking OR tobacco OR cigarette OR gestational cigarette exposure OR gestational smoking OR gestational tobacco exposure OR factor) AND (hypospadias OR congenital anomalies OR genitourinary anomalies). The reference lists of included papers were also searched to find any additional research that may have been overlooked.

### Inclusion criteria

In accordance with the research question (PICO), the following inclusion criteria were established for the selection of articles: studies on women who gave birth to a live-born son (patients, P); studies that examined maternal active smoking/maternal passive smoking/paternal smoking during pregnancy for risk of hypospadias (intervention, I); studies that used no cigarette exposure during pregnancy as a comparison (comparison, C); and studies that described the risk of hypospadias in their offspring (outcome, O).

### Exclusion criteria

Studies were excluded if the following criteria were met: (1) statistical data for case and control groups or effect estimates were unavailable. (2) the absence of a non-smoking control group.

### Data extraction

Two independent reviewers screened the abstracts and titles of the studies retrieved during the search procedure to identify those studies that potentially meet the inclusion criteria. Then, reviewers evaluated the entire text of the identified studies to determine which ones should be included in the study. Disputes were resolved through discussion or consultation with a third author.

The fundamental data from selected studies would be extracted, including the first author, the year of publication, the participants’ country, the study period, the number of hypospadias cases and sample size of cohort studies, or the total sample size of case and control group for case-control studies, the exposure of interest, the assessment method of smoking status, the reported exposure time, the assessment method of hypospadias, whether the confounding factors were controlled, and quality score (Table 1).

**Table 1:** Extracted fundamental information of papers included in the meta-analysis.

Case-control studies									
Study	Participants’ country	Study period	Case/Control	Reported exposure of interest	Exposure time	Assessment method of smoking status	Assessment method of hypospadias	Whether the confounding factors were controlled	Quality score
Sakemi et al. 2022	Japan (Asian)	2010–2014, 2016–2019	43/7,822	MAS	NS	SR	MR	No	6
Siregar et al. 2022	Indonesia (Asian)	2021–2022	60/60	MAS	During pregnancy	SR	MR	Yes	6
Spinder et al. 2021	Netherlands (Europe)	1997–2013	370/5,585	MAS	During pregnancy	SR	MR	No	8
Akay et al. 2021	Turkey (Asian)	2015–2019	280/149	MAS	During pregnancy	MR	MR	No	4
				PAS	NS	MR	MR	No	4
Jamaladin et al. 2020	Netherlands (Europe)	Cases: 1975–2016 Controls: 1990–2011	887/1,005	MAS	During the periconceptional period	SR	MR	No	7
White et al. 2019	America (North America)	1999–2008	8,981/89,910	MAS	NS	MR	MR	No	8
Li et al. 2019	Sweden (Europe)	2001–2010	3,704/497,584	MAS	During pregnancy	SR	MR	Yes	8
Estors Sastre et al.2019	Spain (Europe)	2015–2017	210/210	PAS	NS	SR	MR	Yes	7
Haraux et al. 2018	France (Europe)	2011–2014	25/58	MAS	During pregnancy	SR	MR	No	7
Camichael et al. 2017	America (North America)	1997–2011	1,979/4,342	MAS	In the early pregnancy	SR	MR	Yes	8
				MPS	During the periconceptional period	SR	MR	Yes	8
Mavrogenis et al. 2015	Hungary (Europe)	1980–1996	203/800	MAS	During pregnancy	SR	MR	No	7
Xu et al. 2014	China (Asian)	2003–2012	193/835	MAS	NS	SR	MR	No	6
				PAS	NS	SR	MR	No	6
Winston et al. 2014	America (North America)	2003–2005	995/16,002	MAS	NS	MR	MR	No	7
Woud et al. 2014	France (Europe)	1997–2009	2,148/5,183	MAS	During the periconceptional period	SR	MR	No	7
van Rooij et al. 2013	Netherlands (Europe)	Cases: 1987–1997, 2003–2006 Controls: 1996–2004	405/627	MAS	In the early pregnancy	SR	MR	Yes	8
				PAS	During the periconceptional period	SR	MR	Yes	8
Christensen et al. 2013	Denmark (Europe)	2003–2005	305/306	MAS	During pregnancy	SR	MR	No	7
Rignell-Hydbom et al. 2012	Sweden (Europe)	1986–2002	222/224	MAS	In the early pregnancy	MR	MR	No	8
Adams et al. 2011	America (North America)	1992–2008	2,339/34,904	MAS	During pregnancy	MR	MR	No	7
Iszatt et al. 2011	England (Europe)	2000–2003	471/490	MAS	In the early pregnancy	SR	MR	No	7
Akin at al. 2011	Turkey (Asian)	2007–2008	28/252	MAS	During pregnancy	SR	MR	No	6
Brouwers et al. 2010	Netherlands (Europe)	1996–2004	305/629	MAS	During pregnancy	SR	MR	Yes	7
				PAS	Before pregnancy	SR	MR	Yes	7
Ormond et al. 2009	England (Europe)	1997–1998	2,393/12,465	MAS	During pregnancy	SR	MR	Yes	8
				MPS	In the early pregnancy	SR	MR	No	8
Nørgaard et al. 2009	Denmark (Europe)	1996–2005	468/485	MAS	In the early pregnancy	SR	MR	No	7
Rodriguez-Pinilla et al. 2008	Spain (Europe)	1986–2004	1,591/15,000	MAS	In the early pregnancy	MR	MR	Yes	6
Carbone et al. 2007	Italy (Europe)	1998–2002	133/203	MAS	During pregnancy	SR	MR	No	8
				PAS	NS	SR	MR	No	8
Brouwers et al. 2007	Netherlands (Europe)	1987–1997	583/251	MAS	During pregnancy	SR	MR	No	6
				MPS	Before pregnancy	SR	MR	No	6
Meyer et al. 2006	America (North America)	1998–2002	354/727	MAS	During pregnancy	MR	MR	No	7
Carmichael et al. 2005	America (North America)	1997–2000	453/1,267	MAS	In the early pregnancy	SR	MR	No	8
				MPS	In the early pregnancy	SR	MR	No	8
Porter et al. 2005	America (North America)	1987–2002	2,006/10,084	MAS	NS	MR	MR	No	7
Kurahashi et al. 2005	Japan (Asian)	Cases: 2001–2004 Controls: 2000–2003	24/50	MAS	During pregnancy	SR	MR	No	6
Pierik et al. 2004	Netherlands (Europe)	1999–2001	56/313	MAS	NS	SR	MR	No	8
				PAS	NS	SR	MR	No	8
Akre et al. 1999	Sweden (Europe)	1983–1993	1,137/5,687	MAS	In the early pregnancy	SR	MR	Yes	8
Zhang et al. 1992	China (Asian)	1986–1987	53/1,012	PAS	NS	SR	MR	No	4
Van de Eeden et al. 1990	America (North America)	1984–1986	206/4,323	MAS	During pregnancy	MR	MR	Yes	7
Kallen et al. 1988	Sweden (Europe)	1973–1981	170/315	MAS	In the early pregnancy	SR	MR	No	4

Cohort studies									
Study	Participants’ country	Study period	Case/Cohort size	Reported exposure of interest	Exposure time	Assessment method of smoking status	Assessment method of hypospadias	Whether the confounding factors were controlled	Quality score
Lindbo et al. 2022	Denmark (Europe)	1991–2016	193,562/620,224	MAS	During pregnancy	SR	MR	Yes	9
Yang et al. 2022	America (North America)	2016–2019	844,452/11,282,801	MAS	In the early pregnancy	SR	MR	Yes	8
Kjersgaard et al. 2022	Denmark (Europe)	1989–2012	13,981/63,583	MAS	During pregnancy	SR	MR	Yes	9
				PAS	During pregnancy	SR	MR	Yes	9
Perry et al. 2019	America (North America)	2006–2015	247,939/1,102,890	MAS	In the early pregnancy	SR	MR	Yes	6
Kovalenko et al. 2019	Russia (Europe)	2006–2011	6,300/19,175	MAS	During pregnancy	MR	MR	Yes	7
Ghazarian et al. 2018	America (North America)	1959–1965	10,360/11,965	MAS	NS	MR	MR	No	8
Kallen et al. 2002	Sweden (Europe)	1983–1996	348,228/1,068,845	MAS	In the early pregnancy	SR	MR	No	9
Seidman et al. 1990	Israel (Asian)	1974–1976	2,675/14,477	MAS	During pregnancy	SR	MR	No	7
Shiono et al. 1986	America (North America)	1974–1977	9,269/24,165	MAS	In the early pregnancy	SR	MR	No	7

MAS, maternal active smoking; MPS, maternal passive smoking; MR, medical records; NS, not stated; PAS, paternal active smoking; SR, self-report. Periconceptional period includes the 3 months before pregnancy and first 3 months of pregnancy. Early pregnancy includes the first month, the first trimester, the first 4 month, and the time before the first antenatal visit (13–15 weeks of gestation).

### Assessment of risk of bias

The Newcastle-Ottawa Scale (NOS), the Agency Guidelines for Healthcare Research and Quality (AHRQ), was used to assess study quality and the potential for bias. A star system (range, 0–9 stars) was developed to rate the quality of research in the three categories of participant selection, group comparability, and exposure and result of interest for investigations, respectively. A study with seven or more stars as being low risk of bias was defined. The details are listed in Table 2.

**Table 2:** Evaluation of the quality of included studies (case-control or cohort studies) using the Newcastle-Ottawa Scale (NOS).

Quality assessment of case-control studies										
Reference	Year	Seletion				Comparability	Exposure			Scores
		Adequacy of case definiton	Representativeness of cases	Selection of controls	Definition of controls	Comparability of cases and controls	Ascertainment of exposure	Same ascertainment for cases and controls	Non-response	
Sakemi	2022	*	*	NA	*	**	NA	*	NA	6
Siregar	2022	*	*	NA	*	**	NA	*	NA	6
Spinder	2021	*	*	*	*	**	NA	*	*	8
Akay	2021	*	*	NA	*	NA	NA	*	NA	4
Jamaladin	2020	*	*	NA	*	**	NA	*	*	7
White	2019	*	*	*	*	**	NA	*	*	8
Li	2019	*	*	*	*	**	NA	*	*	8
Estors Sastre	2019	*	*	NA	*	**	NA	*	*	7
Haraux	2018	*	*	NA	*	**	NA	*	*	7
Carmichael	2017	*	*	*	*	**	*	*	NA	8
Mavrogenis	2015	*	*	*	*	**	NA	*	NA	7
Xu	2014	*	*	NA	*	**	NA	*	NA	6
Winston	2014	*	*	*	*	**	NA	*	NA	7
Woud	2014	*	*	*	*	**	NA	*	NA	7
van Rooij	2013	*	*	*	*	**	NA	*	*	8
Christensen	2013	*	*	*	*	**	NA	*	NA	7
Rignell-Hydbom	2012	*	*	*	*	**	NA	*	*	8
Iszatt	2011	*	*	*	*	**	NA	*	NA	7
Akin	2011	*	*	NA	*	**	NA	*	NA	6
Adams	2011	*	*	*	*	**	NA	*	NA	7
Brouwers	2010	*	*	NA	*	**	*	*	NA	7
Ormond	2009	*	*	*	*	**	*	*	NA	8
Nørgaard	2009	*	*	*	*	**	NA	*	NA	7
Elvira	2008	*	*	NA	*	**	NA	*	NA	6
Carbone	2007	*	*	*	*	**	*	*	NA	8
Brouwers	2007	*	*	NA	*	**	NA	*	NA	6
Meyer	2006	*	*	*	*	**	NA	*	NA	7
Carmichael	2005	*	*	*	*	**	*	*	NA	8
Porter	2005	*	*	*	*	**	NA	*	NA	7
Kurahashi	2005	*	*	NA	*	**	NA	*	NA	6
Pierik	2004	*	*	*	*	**	*	*	NA	8
Akre	1999	*	*	*	*	**	*	*	NA	8
Zhang	1992	*	*	NA	*	NA	NA	*	NA	4
Van de Eeden	1990	*	*	*	*	**	NA	*	NA	7
Kallen	1988	*	*	NA	*	NA	NA	*	NA	4

Quality assessment of cohort studies										
Reference		Selection				Comparability	Outcome			Scores
		Representativeness of exposed cohort	Selection of non-exposed cohort	Ascertainment of exposure	Demonstrated outcome was not present at start of study	Comparability of cohorts	Assessment of outcome	Length of follow-up	Adequacy of follow-up	
Yang	2022	*	*	*	NA	**	*	*	*	8
Kjersgaard	2022	*	*	*	*	**	*	*	*	9
Perry	2019	*	*	NA	NA	**	*	*	NA	6
Kovalenko	2019	*	*	NA	NA	**	*	*	*	7
Ghazarian	2018	*	*	*	NA	**	*	*	*	8
Lindbo	2014	*	*	*	*	**	*	*	*	9
Kallen	2002	*	*	*	*	**	*	*	*	9
Seidman	1990	*	*	*	NA	**	*	*	NA	7
Shiono	1986	*	*	NA	*	**	*	*	NA	7

The asterisk (*) symbol means the referred study could get 1 score in corresponding aspect according to Newcastle-Ottawa Scale.

Two asterisk (**) symbol means the referred study could get 2 score in corresponding aspect.

### Statistical analysis

The association between parental smoking and hypospadias in offspring was evaluated using RRs. Due to the low incidence of hypospadias, ORs were regarded as RRs directly. First, we estimated the summary RRs and 95% CIs for the risk of hypospadias associated with parental smoking using the RRs and 95% CIs from each study. Then, using the chi-square test (with a *p*-value of <0.1 and ≥0.1indicating high and low heterogeneity, respectively) and *I*^2^ index statistics (varied from 0% to 100%), we determined the heterogeneity of these studies, which was described as no (0%–25%), low (26%–50%), moderate (51%–74%), and high (75%–100%). When there was evidence of heterogeneity (*I*^2^ > 50%) across studies, random-effects models were used to generate the combined RRs and the corresponding 95% CIs, whereas fixed-effects models were employed otherwise. Subgroup analyses were undertaken based on the study's primary outcomes to investigate the potential causes of heterogeneity: study design, participants’ region by continent, assessment methods of smoking, whether the confounding factors were controlled, smoking exposure time, and quality score.

Furthermore, publication bias was to be examined visually using funnel plots if the number of studies in either cohort equalled or exceeded 10. Stata version 15.1 and the statistical software R 4.1.1 were used to conduct all analyses.

## Results

### Search results and study characteristics

**Figure 1:**
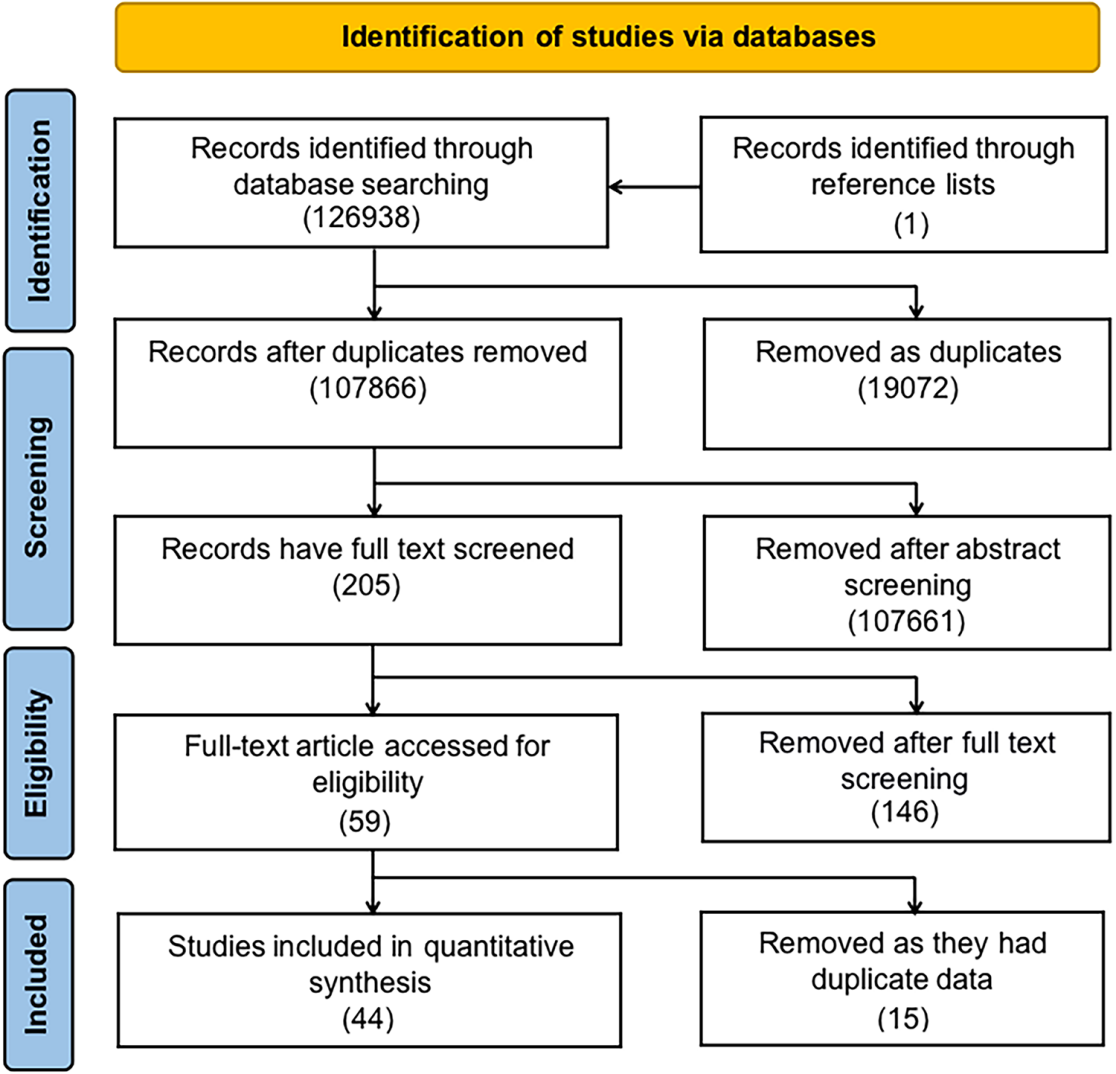
Flow diagram illustrates the identification of studies from the literature and their subsequent inclusion or exclusion from the meta-analysis.

Figure 1 is a diagrammatic illustration of the study identification and inclusion process. 126,938 articles were identified using our systematic search approach in PubMed, the Cochrane Library, EMBASE, and Web of Science. We retained 107,865 articles after removing duplicate studies. Following a review of the titles and abstracts, it was determined that 107,761 papers did not meet the research objective. After comprehensively screening 204 full texts, 58 papers were found to meet our inclusion criteria. Additionally, we searched the reference lists of chosen papers for relevant studies. We found one additional article (9). After reviewing the entire texts of fifty-nine studies, we found that several studies used similar initial data, running the risk of the same patients being included in the meta-analysis twice [Kallen et al. (10) and Kallen et al. (11); Giordano et al. (12) and Carbone et al. (13); Carmichael et al. (14), Carmichael et al. (15), Van Zutphen et al. (16) and Hoyt et al. (17); de Kort et al. (18), Rocheleau et al. (19), and Ormond et al. (20); Rappazzo et al. (21) and Winston et al. (22); Caton et al. (23) and Carmichael et al. (1); Leite et al. (24) and Lindbo et al. (25); Agopian et al. (26), White et al. (27) and Sheth et al. (28); Haraux et al. (29) and Haraux et al. (30); Trabert et al. (31) and Ghazarian et al. (32); Akre et al. (33) and Kjersgaard et al. (34)]. After comparing the quality and methods of these articles, we selected Kallen et al. 1988, Carbone et al. Carmichael et al. 2017a, Ormond et al. Winston et al. Carmichael et al. 2005, Lindbo et al. Agopian et al. Haraux et al. 2018, Ghazarian et al. and Kjersgaard et al. for data extraction, as they more closely met our inclusion criteria and had the lower risk of bias.

Our meta-analysis includes thirteen studies from Hackshaw et al. (6) and nine studies from Zhang et al. (8). Two of the studies included by Hackshaw et al. were excluded. One was excluded due to data overlap (23), while another study was a book, for which data were unavailable (35). Three of the studies included by Zhang et al. were excluded due to data overlap (11, 12, 33).

Table 1 summarizes the included studies’ features, which comprised 16,637,830 participants and were published between 1986 and 2022. 42 studies, 9 studies, and 3 studies reported maternal active smoking, paternal smoking, and maternal passive smoking, respectively. 35 (79.5%) of the 44 papers included in this study employed a case-control design (1, 9, 10, 13, 14, 20, 22, 27, 30, 36–61), whereas 9 (20.5%) employed a cohort study design (25, 32, 34, 62–67).

The quality assessment scores of included studies are summarized in Table 2. Overall, 75.0% (33/44) of these studies received a score higher than 6. The mean value was 6.86 stars for 35 case-control studies and 7.78 stars for 9 cohort studies. The evaluation of the study's quality revealed little evidence of considerable bias that could have significantly impacted the study's outcomes.

### Maternal active smoking and risk of hypospadias

**Figure 2:**
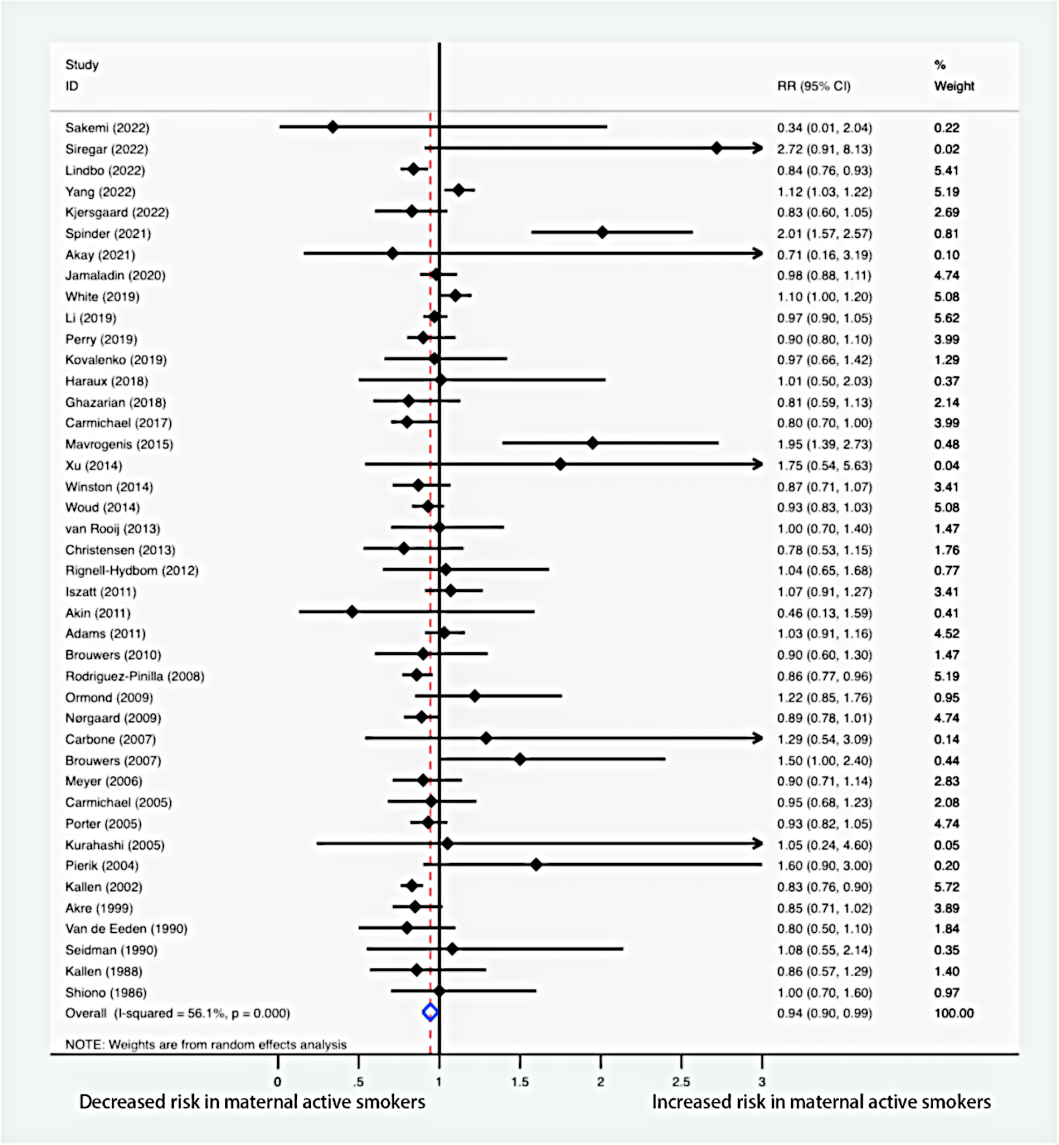
Forest plot for maternal active smoking and risk of hypospadias.

The meta-analysis of the combined RR (Figure 2) indicated that maternal active smoking was associated with a lower risk of offspring hypospadias (RR = 0.94, 95% CI: 0.90–0.99). Moderate heterogeneity between studies led to the use of a random-effects model (*I*^2 ^= 56.1%; *P* < 0.001).

### Paternal smoking and risk of offspring hypospadias

**Figure 3:**
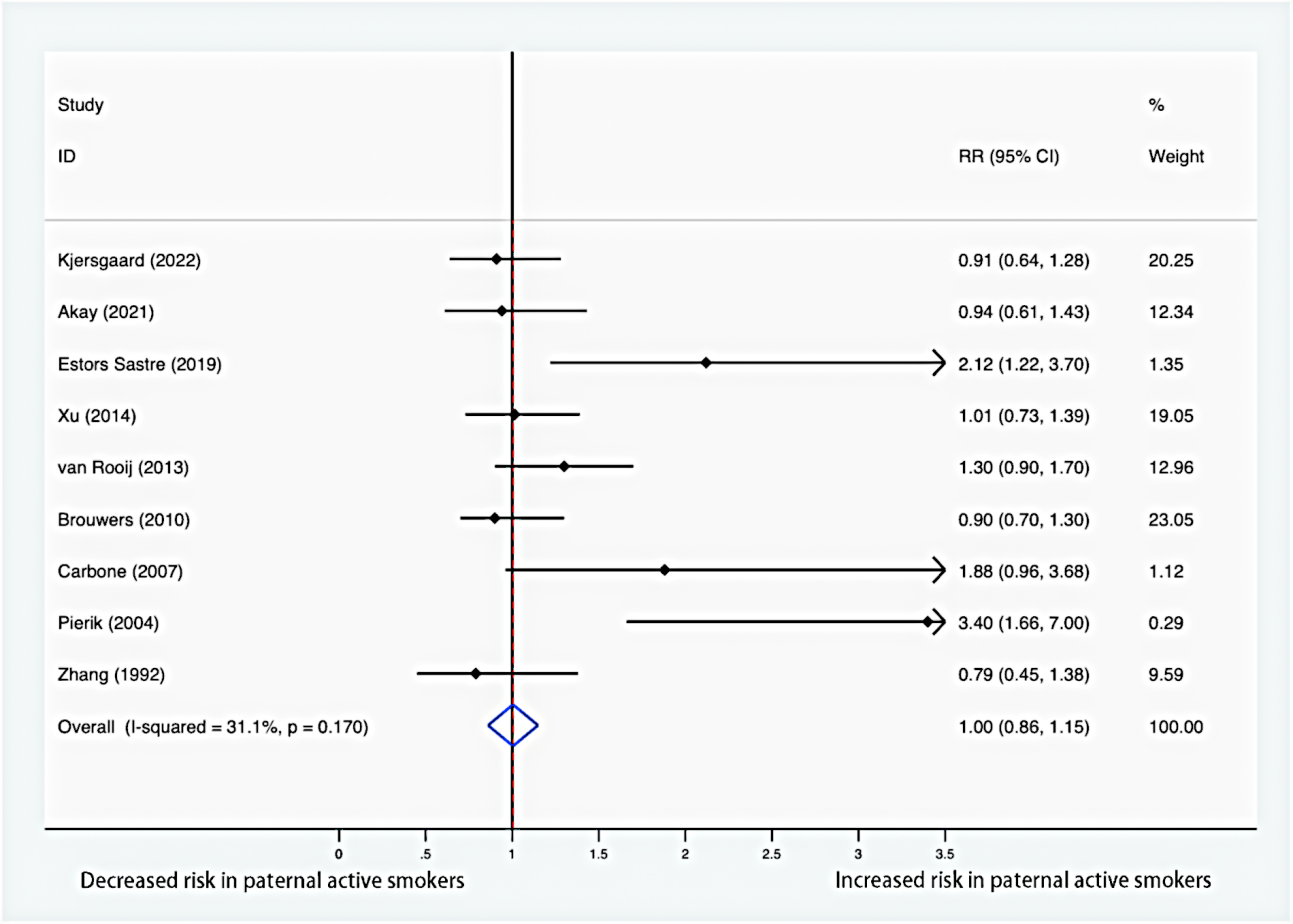
Forest plot for paternal smoking and risk of hypospadias.

Figure 3 summarizes risk estimates between paternal smoking and hypospadias in offspring. There was no significant association between paternal smoking and the risk of hypospadias (RR = 1.00; 95% CI: 0.86–1.15). Moreover, low evidence of heterogeneity was found, and a fixed-effects model was employed (*I*^2 ^= 31.1%; *P* = 0.170).

### Maternal passive smoking and risk of offspring hypospadias

**Figure 4:**
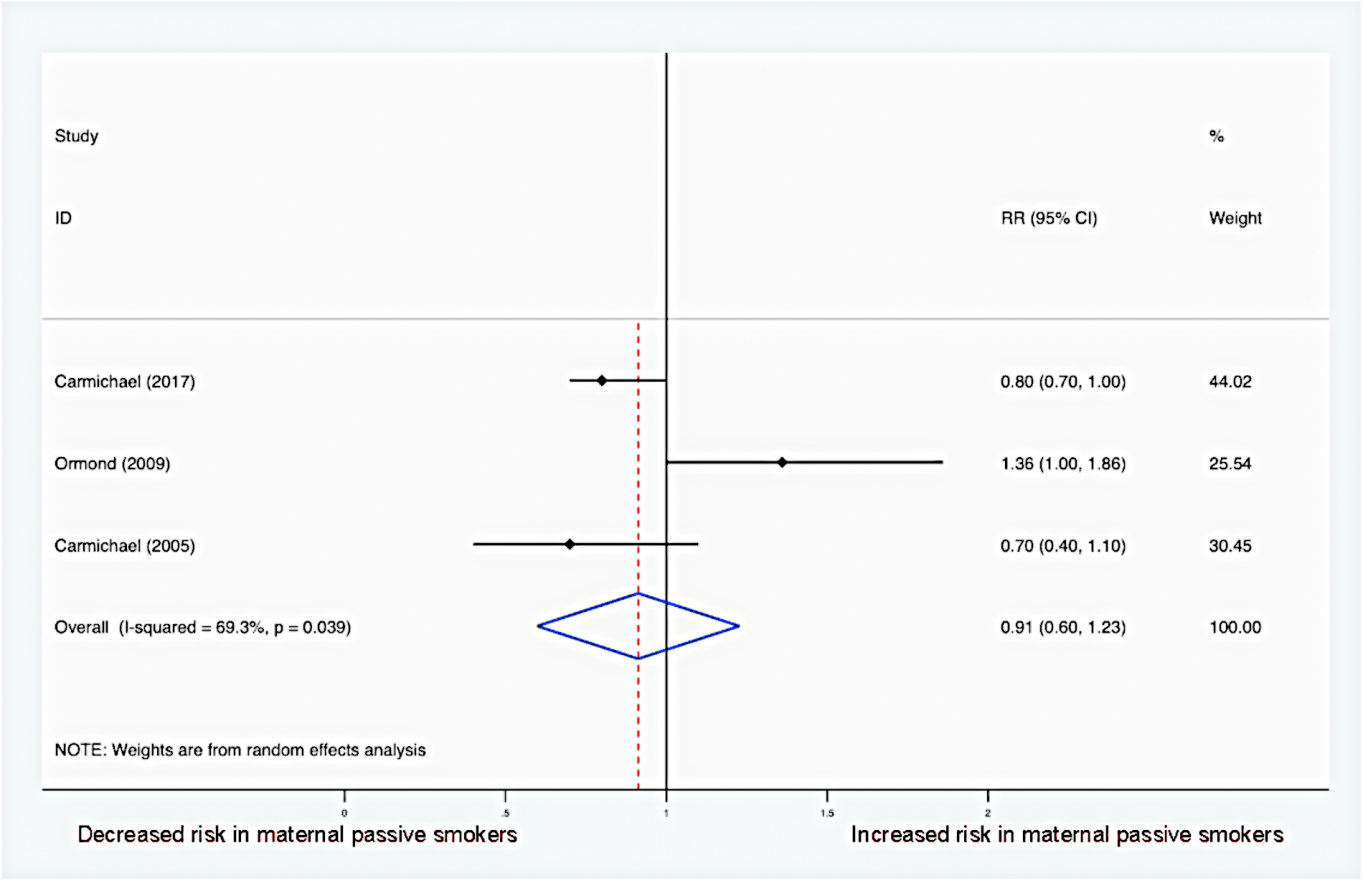
Forest plot for maternal passive smoking and risk of hypospadias.

Figure 4 summarizes risk estimates between maternal passive smoking and hypospadias in offspring. Overall, there was no association between maternal passive smoking and the risk of hypospadias (RR = 0.91, 95% CI: 0.60–1.23). In addition, moderate heterogeneity was detected, and the random-effects model was utilized (*I*^2 ^= 69.3%; *P* = 0.039).

### Subgroup analysis

We performed a subgroup analysis based on the risk of hypospadias and smoking exposure time to determine whether smoking exposure time was the source of heterogeneity (Figure 6). However, no differences were identified (*P *= 0.804; Figure 6).

**Figure 5:**
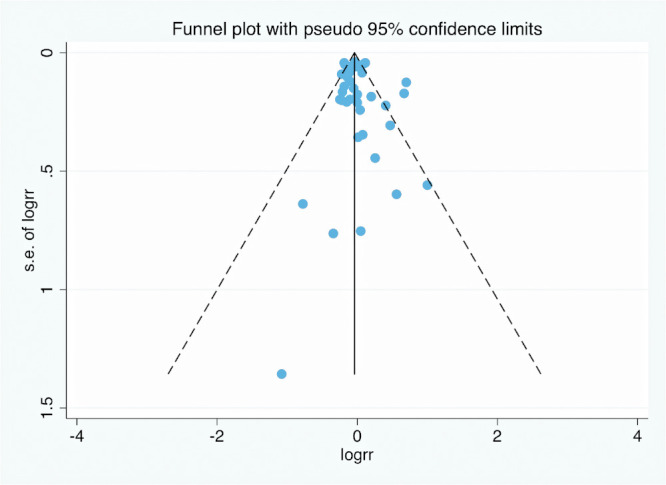
Funnel plot of the risk of hypospadias associated with maternal active smoking.

**Figure 6:**
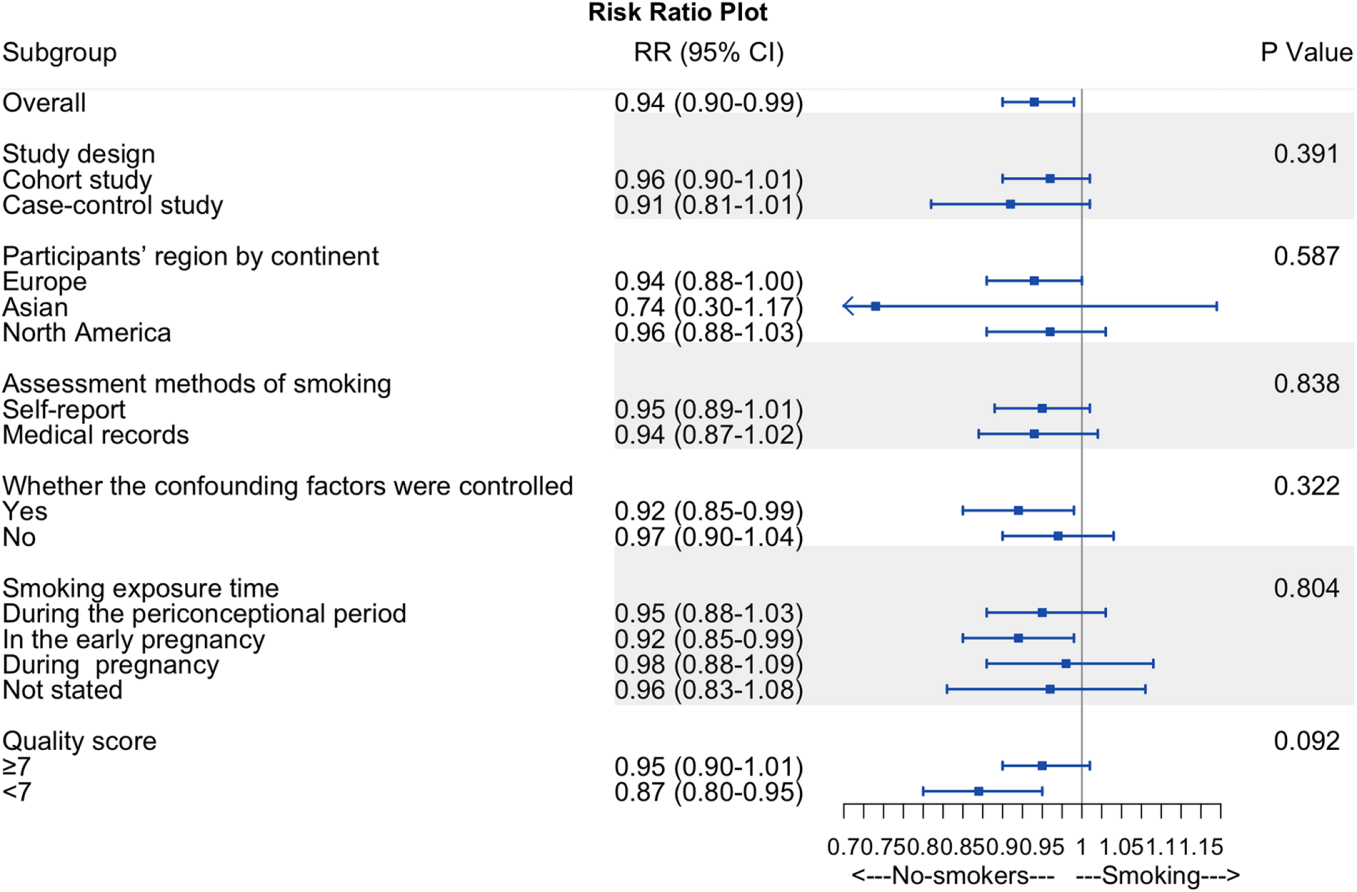
Summary forest plot of subgroup analyses. Summary forest plot of subgroup analyses, including study design, participants’ region by continent, assessment methods of smoking, whether the confounding factors were controlled, smoking exposure time, and quality score.

Other subgroup analyses of maternal active smoking failed to explain the overall analysis's heterogeneity and revealed consistent findings within subgroups (Figure 6). We found no difference in RR between cohort and case-control studies, participants’ region by continent, the assessment methods of smoking, quality scores, and whether the confounding factors were controlled.

### Publication bias

The funnel plot (Figure 5) indicated no evidence of publication bias in risk estimates for maternal active smoking and offspring hypospadias.

We did not analyze publication bias in risk estimates for paternal smoking or maternal passive smoking using a funnel plot because the number of included studies was less than 10.

## Discussion

There is considerable debate over the association between paternal smoking and offspring hypospadias. Our study demonstrated an association between maternal active smoking and a decreased risk of hypospadias in offspring. However, no association was found between maternal passive smoking or paternal smoking and the risk of offspring hypospadias.

Our subgroup analysis showed that the risk of offspring hypospadias was consistent regardless of whether the mother smoked in early pregnancy or during the entire pregnancy, which accorded with the results of a prior Danish study. Eighty-eight percent of pregnant women who smoked at the 16th week continued to do so throughout their pregnancy (68). Consequently, even if some studies collected data on smoking during early pregnancy, it could be a good approximation of exposure status throughout pregnancy.

There are two meta-analyses examining the association between maternal smoking and the risk of hypospadias in offspring (6, 8). However, no meta-analysis has evaluated the risk of hypospadias related to maternal passive smoking or paternal smoking. The 15-study meta-analysis by Hackshaw et al. reported an odds ratio (OR) of 0.90 (95% CI: 0.85; 0.95) for having offspring with hypospadias (6), whereas 12-study meta-analysis by Zhang et al. reported an odds ratio (OR) of 1.16 (95% CI: 1.01; 1.33) for having offspring with hypospadias. Our study has some significant advantages over the previous meta-analysis; for example, we included more existing articles with a total of 16,637,830 participants to more precisely estimate the association between parental smoking and the risk of hypospadias in offspring. We also performed the first meta-analysis on the association between maternal passive smoking and paternal smoking and the risk of offspring hypospadias. Additionally, we performed subgroup analyses to explore potential sources of heterogeneity.

The specific mechanisms underlying the association between maternal smoking and hypospadias in offspring remain unknown and require further study, but various hypotheses based on epidemiological and animal data have been offered. One hypothesis is that a higher androgenic steroid level in the fetuses of pregnant smokers promotes normal urethral closure. According to a prior study, estrogens may inhibit the activity of fetal androgens (69). Smoking may have anti-estrogenic effects by increasing estradiol metabolism to metabolites with little estrogenic activity *via* increased 2-hydroxylation (70), which is consistent with the finding that smoking women have a higher androgenic steroid level (71, 72). In addition, smoking reduces the activity of 21- or 11-hydroxylase in the adrenal cortex, increasing adrenal androgen secretion (73, 74). A higher androgenic steroid level could compensate for intrinsic fetal endocrine testicular insufficiency, fetal androgen receptor deficiency, or decreased fetal 5-reductase activity (65), which promotes normal urethral closure, in fetuses of pregnant smokers.

Our study should also be viewed with certain limitations. First, only one study indicated the exclusion of passive smokers from the comparison of active smokers and no-smokers, as well as the exclusion of active smokers from the comparison of passive smokers and no-smokers (14). The imprecision of the categorization of included studies may introduce bias into the study's findings. Second, there were a limited number of studies on maternal passive smoking, which may have led to biased results. The relationship between maternal passive smoking and the risk of hypospadias requires additional research. Third, cigarette smoking is associated with an increased risk of the majority of congenital defects (6). Our study did not assess the association between smoking and other congenital defects, which may have affected the study's credibility.

In conclusion, the purpose of our study, which included a significant number of participants to ensure statistical power, was to examine the association between parental smoking and hypospadias. Our study demonstrated an association between maternal active smoking and a decreased risk of hypospadias. No association was identified between parental smoking or passive smoking and the risk of hypospadias in offspring. However, quitting cigarettes before pregnancy indeed has a good effect on the health of the offspring and should be advocated worldwide.

## Data Availability

The original contributions presented in the study are included in the article/Supplementary Material, further inquiries can be directed to the corresponding author.
